# Analysis of Smart Lung Tumour Detector and Stage Classifier Using Deep Learning Techniques with Internet of Things

**DOI:** 10.1155/2022/4608145

**Published:** 2022-09-13

**Authors:** Shubham Joshi, Shraddha Viraj Pandit, Piyush Kumar Shukla, Atiah H. Almalki, Nashwan Adnan Othman, Adnan Alharbi, Musah Alhassan

**Affiliations:** ^1^Department of Computer Engineering, SVKM'S NMIMS MPSTME, Shirpur Campus, Shirpur 425405, India; ^2^Department of Artificial Intelligence & Data Science, PES Modern College of Engineering, Pune 411005, Maharashtra, India; ^3^Department of Computer Science & Engineering, University Institute of Technology, Rajiv Gandhi Proudyogiki Vishwavidyalaya (Technological University of Madhya Pradesh), Bhopal 462033, Madhya Pradesh, India; ^4^Department of Pharmaceutical Chemistry, College of Pharmacy, Taif University, P.O. Box 11099, Taif 21944, Saudi Arabia; ^5^Addiction and Neuroscience Research Unit, College of Pharmacy, Taif University, Al-Hawiyah, Taif 21944, Saudi Arabia; ^6^Department of Computer Science, College of Science, Knowledge University, Erbil 44001, Iraq; ^7^Department of Clinical Pharmacy, College of Pharmacy, Umm Al-Qura University, Makkah, Saudi Arabia; ^8^University of Development Studies, Electrical Engineering Department, School of Engineering, Nyankpala Campus, Tamale, Ghana

## Abstract

The use of artificial intelligence (AI) and the Internet of Things (IoT), which is a developing technology in medical applications that assists physicians in making more informed decisions regarding patients' courses of treatment, has become increasingly widespread in recent years in the field of healthcare. On the other hand, the number of PET scans that are being performed is rising, and radiologists are getting significantly overworked as a result. As a direct result of this, a novel approach that goes by the name “computer-aided diagnostics” is now being investigated as a potential method for reducing the tremendous workloads. A Smart Lung Tumor Detector and Stage Classifier (SLD-SC) is presented in this study as a hybrid technique for PET scans. This detector can identify the stage of a lung tumour. Following the development of the modified LSTM for the detection of lung tumours, the proposed SLD-SC went on to develop a Multilayer Convolutional Neural Network (M-CNN) for the classification of the various stages of lung cancer. This network was then modelled and validated utilising standard benchmark images. The suggested SLD-SC is now being evaluated on lung cancer pictures taken from patients with the disease. We observed that our recommended method gave good results when compared to other tactics that are currently being used in the literature. These findings were outstanding in terms of the performance metrics accuracy, recall, and precision that were assessed. As can be shown by the much better outcomes that were achieved with each of the test images that were used, our proposed method excels its rivals in a variety of respects. In addition to this, it achieves an average accuracy of 97 percent in the categorization of lung tumours, which is much higher than the accuracy achieved by the other approaches.

## 1. Introduction

Recently, a significant number of people all across the world have become ill with the pandemic illness known as COVID-19. E-diagnosis, remote access, virtual consultants, and e-treatment have all made their way into the healthcare industry as a result of the current climate, which has led to the elimination of the need for physical personalization and a reduction in the risk of disease transmission. It has taken the healthcare business to a new degree of severity, which has resulted in an increase in the mortality rate of people suffering from chronic illnesses, in particular those who are afflicted with cancer, diabetes, and cardiovascular diseases. This is because there is a scarcity of medical professionals, including doctors, nurses, and radiologists [1], which has led to an increase in the number of people diagnosed with cancer. The number of people who pass away as a result of cancer and other chronic diseases is continuing to climb at an alarming rate every year throughout the whole world, especially in less developed nations. According to WHO [2], in 2010, lung cancer was the largest cause of mortality due to the disease, accounting for 1.80 million fatalities, or 18 percent of all cancer-related deaths. A disproportionate increase in transitioning countries (from 65 percent to 94 percent) versus transitioned countries (33 percent to 57 percent) is expected due to demographic change. Despite this, the global tumour burden is expected to reach 28.40 million cases in 2040, which is a 48 percent increase from 2020. This will be further exacerbated by an increase in risk factors such as smoking. Standard challenges on recognising lung tumours in patients from decade include zero symptoms that are not related to age factor, patients who have breathing problems, patients who have smoked for 30–40 years, and patients who have no symptoms[3, 4]. Numerous researchers have used a wide array of methodologies, including segmentation, detection, and classification techniques; in an effort to circumvent the challenges that are associated with the diagnosis of lung cancer [5, 6], artificial intelligence has played an increasingly since it was first introduced. This is due to the fact that artificial intelligence is suitable for solving these types of problems. The artificial intelligence-based supervised learning models that Pragya and her colleagues utilised in order to detect lung tumours and classify them as either malignant or benign can be found in [7]. The multilayer preceptor, support vector machine (SVM), and key-value network (KNN) classifiers were developed as binary lung tumour classifiers, as stated by Rodriguez et al. [8]. Dinesh and his colleagues devised a grey wolf optimization approach, which was then combined with a genetic algorithm in order to create a hybrid lung tumour classifier [9]. This was done in order to better understand how genetic algorithms work. Traditional methods, on the other hand, have a number of shortcomings, among which are the facts that they are insufficient for early detection, that they are less efficient in terms of accuracy rate, and that they are not suitable for stage categorization.

The construction of a DCNN consists of a four-layer design with a ReLU activation function [9], as can be seen above. The researchers Zhuoliu et al. created a reinforcement Q-learning system for the detection of tumours. The system classified tumours as either malignant or benign according on the stage of their growth. The author states that the challenges that arose during the process of creating the RNN model for the detection of lung cancer in terms of its localization were resolved [10].

**Figure 1:**
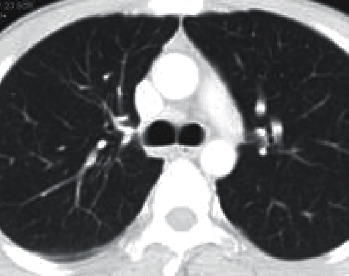
Input image.

We were encouraged by the results that these novel strategies produced, and as a result, we made the decision to use them in the healthcare industry [11]. As a result of our research, we were able to develop a new prototype algorithm that we call the Smart Lung Tumour Detector and Stage Classifier (SLD-SC). This algorithm is able to detect lung cancer at the earliest possible stage by utilising information obtained from PET scans (Figure 1).

Our group came up with the concept for an intellectual diagnostic module that they called SLD-SC in an effort to reduce the overall mortality rate as well as to improve their ability to identify lung tumour cells caused by non-small-cell lung cancer (NSLC). Unsupervised learning methods are used in the lung cancer detector that was proposed. These algorithms are used for segmentation, feature extraction, and stage classification. In addition to the Internet of Things, there are cloud servers where databases may be stored. The diagram labelled “Figure 1” is an example of the entire system that is being discussed.

- The LIDC CT DICOM images and PET scans are analysed to start, with the goal of determining the amount of noise that is now present, as well as the quantity of memory that is necessary, and so on.
- A modified version of the LSTM model has been constructed with the intention of pinpointing the parts of the lung tumour that are most specific and accurate. These zones have been segregated from one another and are now being utilised for categorization purposes in order to establish an appropriate level of lung cancer severity.
- This method makes use of multilayer convolutional neural networks, which are abbreviated as M-CNN, in order to efficiently categorise the various phases of tumour development.
- The findings of a range of tests that were carried out with the aid of numerous medical datasets are presented in this article. These experiments were carried out by the authors of this paper. In addition to this, we make use of real-time data that was obtained patients by way of an Internet of Things device.
- The recommended SLD-SC has been carried out in order to carry out performance measures for a new technique, which may also be found in this work. These measurements have been carried out in this study.

The structure of the paper may be broken down into the following outline: In the second section, we looked at and spoke about the relevant literature; in the third section, we concentrated on the technique and went into further detail about it. The reasoning that underpinned this method was deconstructed and put into the appropriate context.

## 2. Related Works

Alnuaim et al. developed the unsupervised learning model known as 3D Alex Net [12]. The suggested Alex Net detection technique is put to the test using the LUNA dataset. The proposed model is inefficient because there is insufficient testing data; just 10% of the training database is being utilised. This results in an inability to accurately predict outcomes.

Tafadzwa et al. developed a supervised CNN predictor with the purpose of identifying individuals with lung cancer who were in the early stages of adenocarcinoma (ADC) and squamous cell carcinoma (SCC). Validation of CNN was performed using real-time data from non-small-cell lung cancer patients obtained at Massachusetts General Hospital from patients in the early stages of the disease [13, 14].

Reference [14] According to the findings of the testing, the accuracy of the suggested approach was measured at 90.85 percent [15]. Ruoxi et al. detailed the process of determining the presence of EGFR mutations with the use of computer-assisted diagnostics. This process involves obtaining, analysing, and fusing many types of interdependent characteristics [16]. This research makes use of an innovative hybrid network model that is constructed using CNN and RNN architectural components. The CNN algorithm is used to extract the quantitative aspects of an image, and the LSTM algorithm is used to describe the connection between the various kinds of features [17].

According to their results, multitype dependency-based feature representations performed much better than single-type feature representations (accuracy of 75%, area under the curve = 0.78) when compared to traditional features that were extracted [18]. This strategy was developed in order to classify the various types of cancer that can be caused by tumour RNA sequences found in genomic data (CNN). In this particular research [19], the performance metrics that were discussed were recall, precision, and *F*1-score. According to Abdulgani et al., label-free techniques do not cause any damage to cells and do not result in any changes to the makeup of cells or their innate characteristics. The objective of this study was to enhance cell categorization by using observed optical profiles, and it did so by combining recent breakthroughs in optical measurements with Prony's techniques [20]. He and his colleagues were able to locate signature genes via the development of more accurate Tobacco Exposures Pattern (TEP) Classification models and the discovery of the interaction connections between those models on many biological levels [21]. The most current models and datasets that have been used for the execution of a variety of algorithms are summarised in Table 1.

## 3. Proposed Methodology

**Figure 2:**
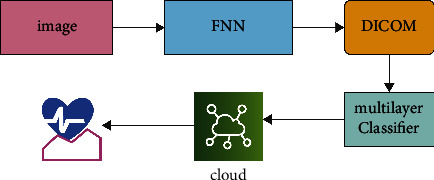
Proposed work framework.

The proposed SLD-SC hybrid framework consists of lung tumour detector, lung tumour segmentation, and stage classifier modules. These modules allow for correct results to be achieved in terms of “accuracy, precision, and recall.” Figure 2 provides an overview of the proposed model, which is then followed by descriptions.

### 3.1. Outline of Proposed Work

#### 3.1.1. Input Image

A PET scan was performed every two to three minutes in each of the eight or nine different bed positions. A three-dimensional iterative reconstruction approach was used to piece the photographs back together after they had been destroyed. Every individual who took part in the research was given the chance to provide their informed permission. 7 female patients and 92 male patients were present in the hospital [22]. Up to this point, there has been no study done on the differences in tumour variability between male and female NSCLC patients. As a consequence of this, while we were developing this research, we did not investigate the impact of gender on the specific features of the various cancer subtypes. It was discovered that 45 individuals were suffering from an ADC, whereas the other patients were diagnosed with SqCC. The data collection performed by the Lung Image Database Consortium (LIDC-IDRI) was responsible for providing the DICOM CT lung images [23]. Every single DICOM lung CT scan is recorded in the DICOM file format, which has a dimension of 512 by 512 pixels and is used to store the data. This data collection contains pictures of tissue slices that range from 0.45 millimetres to 0.75 millimetres in diameter and 1.15 millimetres to 2.75 millimetres in thickness. Each radiologist independently reviewed every CT scan and assigned a label to each lesion, based on which of the following three groups it belonged to: nodules, seminodules, or nonnodules. The different forms of tumours were determined after the examination of the CT images by four radiologists [24].  Table 2 represents the patient ID with stages.

Selected nodules were characterised as “well-circumscribed, juxta-vascular, juxta-pleural, pleural-tail.” The ACM method centred its attention on a location of interest (a lung tumour) that had a nodule size of (75 × 85 × 45) millimetres [25]. It is possible to calculate a few radiologists stated the number of voxels that were engaged across all dimensions and extract information on the size of the nodule, as shown in Table 2. The features of both the genuine and the simulated nodules are mentioned in Table 2.

### 3.2. Lung Tumor Detection Using Modified LSTM Model

Applications that need text recognition and voice processing are probably the ones that make use of RNN because of the storage capacity it offers [11]. The process of transferring data is carried out from one state to the next inside the network in a sequential method. The limitations of this recurrent neural network include making it inappropriate for lengthy sequence prediction, and it also suffers from vanishing gradient corruption [26]. Connected cells and gates form the LSTM's memory block, which is made up of the LSTM's building blocks. These gateways and cells are used rather often for the goals of retaining input states and updating [27].  Table 3 represents the LSTM features [28].

LSTM performs better than conventional RNNs due to the distinctive nature of its memory. The structure of an LSTM is shown below, along with a mathematical representation of it. The memory blocks are separated into three categories [29]: (i) the initial loading gate (), (ii) the final output gate (), and (iii) the middle inspection gate (), with equation 3 representing the activation function.

(1) $$\begin{array}{c} \alpha \left(a\right)=\frac{1}{1+e^{-x}},\alpha \left(a\right)\in \left\{0,1\right\}. \end{array}$$

**Figure 3:**
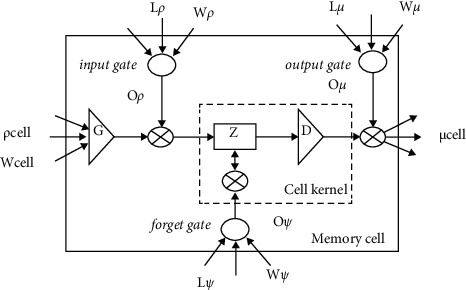
Structure of LSTM network.

The architecture of LSTM blocks may be observed in Figure 3, along with the training function, which is indicated by *q_N_*, and used for executing the equation that describes the entrant states and block values. The kernel functions, which are defined by (6) and (7), are as follows. The structure of the model is represented as a combination of three blocks, and this combination is indicated as “E N (E blocks; )”. The entrant signals of the initial blocks are followed by various states once the model has been constructed. Between the two different units, *u* and *r*, the weights of the strata are denoted by the notation “Wu, r”. When the loading signals are interfaced with the gates at time ‘*j*,' the resulting signal, which also includes the outcome and the feedback signals, is indicated as *E* N (*j*). The indications of the various entrance places are listed down below.

(2) $$\begin{array}{c} E_{N}\left(j\right)=\left(q_{N}\left(j\right)\right),N\in \left\{\delta ,\alpha ,\emptyset \right\}, \end{array}$$

(3) $$\begin{array}{c} P_{N}\left(j\right)=\sum_{y}^{x}zW_{N}*E_{N}\left(j\right),N\in \left\{\delta ,\alpha ,\emptyset \right\}. \end{array}$$

The entrant block is represented as “*δ* block (*j*-1)” and as “Wblock (*j*-1)” called weights and equation.

(4) $$\begin{array}{c} q_{C_{v}^{h}}\left(j\right)=\sum_{y}^{x}zWblock*\delta blocks\left(j\right). \end{array}$$

The product of incoming signals from cells with loading and overlook gates, as well as previous state information, is used to calculate the internal state of the blocks.

(5) $$\begin{array}{c} RC_{v}^{h}\left(j\right)=\;\left\{\begin{array}{c} t=0,\;\emptyset_{a,k}\left(j\right)*R_{C_{v}^{h}}\left(j-1\right)+\;\emptyset_{in,k}\left(j\right)\;.\;E\left(q_{C_{v}^{h}}\left(j\right)\right),\;j>0 \end{array}\right\}, \end{array}$$

(6) $$\begin{array}{c} \gamma \left(a\right)=\frac{4}{1+\;e^{-a}}-2 \end{array}$$

“*ϑ*_*C*_*v*_^*h*^_(*j*)” denotes the output cell block “*C*_*v*_^*h*^” at the sampling time “*j*” which is calculated as follows:

(7) $$\begin{array}{c} \vartheta_{C_{v}^{h}}\left(j\right)=\emptyset_{{\text{out}}_{j}}\left(j\right)*\left(\gamma_{C_{v}^{h}}\left(j\right)\right), \end{array}$$

(8) $$\begin{array}{c} \alpha \left(a\right)=\text{tanh}\left(\frac{a}{2}\right),h\left(a\right)\varepsilon \left\{-1,1\right\}. \end{array}$$

DicomCT lung imaging makes it more difficult to detect nodules in the lungs than it does in other organs, such as the brain. This makes it more challenging to identify lung nodules.

1. If the lung parenchyma was not successfully restored, the patient's mediastinum and thoracic wall will need to be removed from their thorax.

Step two involves the use of the Active Contour Model to segment the part of the lung picture that represents the tumour (ACM) [30].

### 3.3. Lung Tumor Segmentation

**Figure 4:**
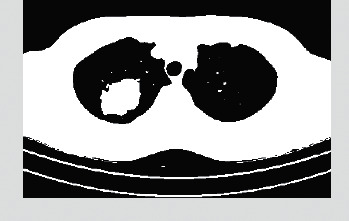
CT image after preprocessing.

**Figure 5:**
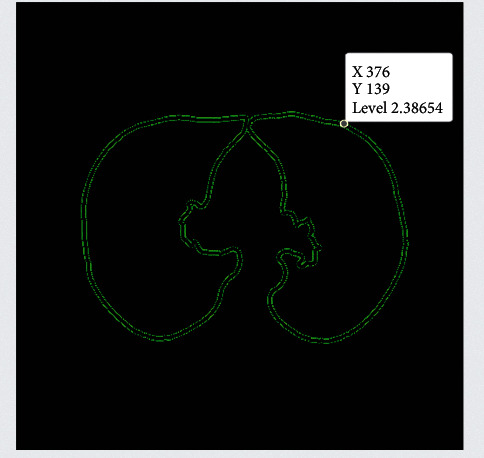
Segmented input image dataset.

The data were used to generate a curve that aids in the identification of tumour sections in the relevant photographs [31]. This curve was then applied to the data. The Snake model was used to create the curve. It is necessary to begin by drawing the curve around the item that has been provided, and then it is necessary for the curve to move its location towards the interior of the object before coming to an end at the object's limits [32]. The proposed method generates three-dimensional features for the CNN classifier by combining two-dimensional stochastic characteristics with three-dimensional data [33]. These three-dimensional features are then fed into the CNN classifier. The segmentation image that was produced from a PET scan as a result of the segmentation method is shown in Figure 4. In the dataset provided by LIDC-IDRI, we were successful in separating out the tumour component, as can be shown in Figure 5.

### 3.4. Input Validation of the Proposed Work

**Figure 6:**
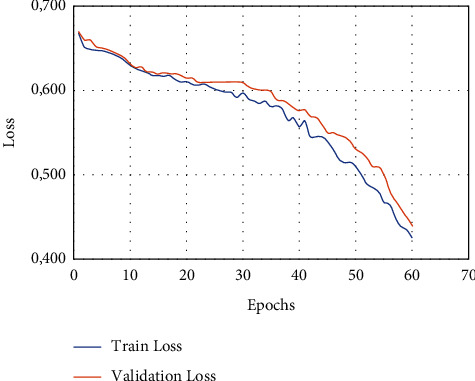
Overall proposed structure performance analysis 1.

As can be seen in Figure 6, the authors of this study make heavy use of the 10-fold cross-validation technique for the LIDC-IDRI/PET dataset. This study made use of the dataset after it had been randomly segmented into stratified 10-fold cross validation. The dataset is one that has been utilised in a great deal of research that is based on deep convolutional neural networks as well as traditional machine learning techniques applied to bioimages and biosignals [34]. A high-performance computing system was developed by the use of a personal computer equipped with an NVIDIA GeForce GTX 1650, a Deep CNN model that was trained from the ground up, along with some random weight (HPC). The number of epochs that will be used for each training phase will be 150 from the dataset that has been provided, and early stopping procedures have been used so that the dataset does not get overfit [35]. In addition, the accuracy analysis with relation to epochs is shown in a very straightforward manner in Figure 6, which covers both the training data and the validation data.

**Figure 7:**
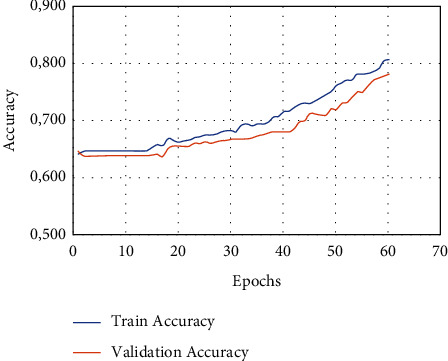
Overall analysis of performance.

Figure 7 shows the performance metrics of the proposed work.

### 3.5. Lung Tumor Stage Classification

**Figure 8:**
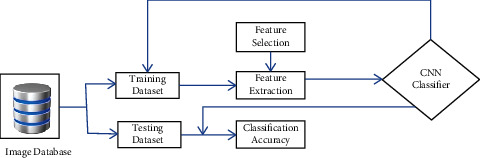
Classification based on image database.

The human brain is used to stimulate artificial neural networks, which enables machine learning to be applied to the solution of complicated issues [36]. Deep learning is one of the subfields that fall under the umbrella of machine learning (DL).  Figure 8 shows the classification-based image database.

**Figure 9:**
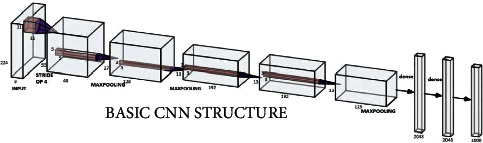
Basic CNN structure.

The DL method is used for the task of extracting characteristics from massive volumes of data; using DL algorithms to glean useful information from massive amounts of data is advantageous in a number of different ways [36]. Because identifying a feature takes a significant amount of time and may be quite expensive, learning-related applications of DL methods do not need the usage of labelled data in any way. In the context of healthcare, we could have both labelled and unlabelled kinds of data, such as X-ray photos taken regardless of the patient's medical state [37], enormous volumes of data that are not labelled.  Figure 9 shows the basic CNN structure.

There are various different deep learning strategies available for your selection. In this part of the article, we discussed some of the most often used examples among them. (1) An artificial neural network, also known as an ANN, is a method for deep learning that consists of a multitude of hierarchical layers and uses perceptrons [38], which are essentially neurons, as its fundamental building pieces. There are many layers used, starting with the input layer and going to the hidden layer, which performs the functions of both the training layer and the output layer. The process begins with the input layer. It is conceivable that the results will not be improved even if the number of concealed layers is greatly increased. This is one of the possibilities [39]. Overfitting may also occur if a high number of layers are added all at once. This can lead to a huge number of distortions, which can provide an excessive amount of interference in the data that is being collected. This can cause overfitting. Up until this moment, the convolutional neural network, also known as a CNN (Figure 7(a)), has been the most recognised example of a useful technology in the field of healthcare. A flight path that has a static extent is being used here as an input [40]. It is possible to utilise it to process medical data, such as image processing for the diagnosis of lung tumours, for instance. It is feasible to connect a large number of perceptrons together and give each of them a weight that is capable of being changed after each iteration of the algorithm [41]. A network is said to be feedforward if waves only go in one direction through it, from the input layer all the way to the output layer. This means that the feedforward network only has one direction in which waves may travel [42].

**Figure 10:**
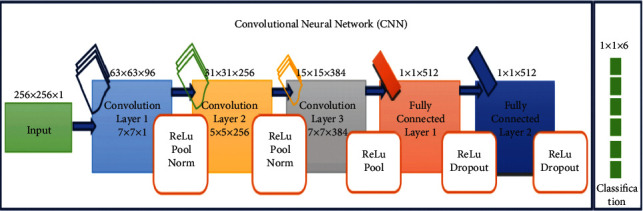
Architecture of the proposed technique.

Figure 10 shows the architecture of proposed technique. It is also one of the deep learning approaches that is used the most frequently. The fact that it is classified as a feedforward network while having several layers indicates that its operation proceeds in just one direction, namely, from input to output [43]. The transmission of data via the process layer results in the extraction of useful features from the input data, which are then shown in the output layers as a direct consequence of the extraction. In the field of medicine, it is used to the process of diagnosing sickness based on samples of tissue collected from patients. Read structures, which are sometimes tough to interpret by human medical professionals, are encouraged to be used as a result of this. Figure 10 is a representation of the proposed architecture for the CNN, which, when implemented, would result in the classification of lung tumour stages based on the data obtained [44].  Table 4 shows the tumor stages.

When compared to other types of tumours, the staging method that is used for lung tumours is what distinguishes them [45]. The techniques that are used for determining the stage of a lung tumour are, for the most part, defined by the specific experiences of doctors as well as the general agreement of the medical community found in both Tables 4 and 5.

### 3.6. IoMT Framework

**Figure 11:**
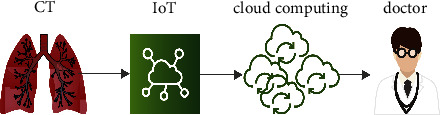
Virtual monitoring and E-diagnosis framework.

An IoMT-based lung tumour identification and stage classification approach is discussed in this part of the study. This methodology makes use of deep learning algorithms to predict the lung tumour picture that has been provided by the public and an end user.  Figure 11 shows the Internet of things architecture.

The Internet of Things (IoT) devices are networked with one another in the field of computer vision for the purpose of data transfer across a network. This calls for interaction between people and computers, in addition to contact between individuals on the human side. The connection provided by the Internet of Things (IoT) is seeing meteoric expansion in the realm of healthcare. This endeavour will not only be useful to patients, but also to the doctors who treat those patients. For instance, with the assistance of a connection to the Internet of Things, patients can get preventative advice from their doctor without having to see him or her, and they can also submit real-time data to their doctor for improved treatment without having to pay a visit. Both of these benefits are made possible without the patients having to physically see their physician. Both of these advantages are available to patients without the prerequisite of their having to schedule an appointment.  Figure 11 illustrates how data may be gathered and transferred between devices that are located in different locations by using sources that are accessible, such as networks and sensors. The diagram is a useful tool for illustrating this procedure. The Internet of Things (IoT) is an essential technology that at the moment has the potential to be employed in the administration of remote medical care. The Internet of Things (IoT) is a network of linked devices that may be worn or implanted and are powered by lightweight and tiny batteries. These devices may also communicate with one another through the Internet. It distributes the information that was gathered by sensors and transmitted through the network to medical institutions, such as hospitals and clinics, in addition to sending it to other sites. It is vital that the data be safeguarded while simultaneously making it accessible to all relevant parties since the existence of this data, which is developing at a rapid pace and may be referred to as “big data,” making it necessary for the data to be protected. The data will be shared in an efficient way by making use of an intelligent and secure architecture, which will be put into place at a variety of medical institutions. This will ensure that the data is kept confidential. Figure 4 depicts the comprehensive structure of the system as a whole, which is based on block chains and makes use of cloud storage to store electronic medical records (EMRs) and other types of information. The information that was acquired by instruments is first delivered to a PDA device under the architecture that we propose. After that, the PDA device will produce the hash of the medical information by making use of the typical hash algorithms. At this point, the hash will be uploaded to a private block chain link that is accessible through the Internet. Everyone involved in the healthcare industry, like as hospitals, insurance companies, laboratories, and other organisations, will function as a block-chain node to enable the free flow of information. This includes patients, doctors, and lab technicians. This includes hospitals, clinics, and research centres as well as other types of labs. Hash is being directed by a PDA device; it will be recognised by every node in the network. This data block has to be validated and verified with the help of the nodes so that the hash can be identified. The authentication operation is carried out on the basis of the hash that has already been established, and after that, as is customary, the hash of the most recent data block is compared to the hash that has already been established. It is possible that the hash of the most recent block of data generated by the PDA device will be included in the block of data that is produced by the PDA device. This is something that is a possibility. There is a possibility that this will take place. The overwhelming majority of the nodes that comprised the block chain required authenticating blocks before they could participate.

**Figure 12:**
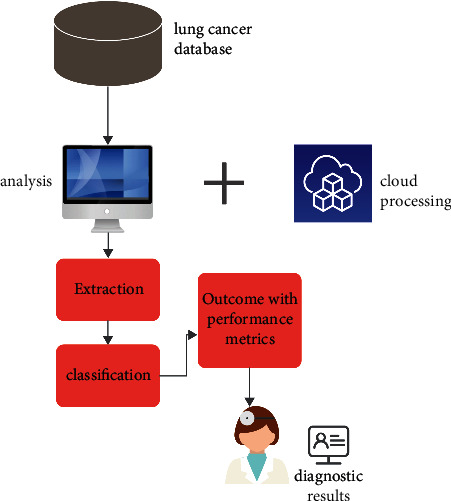
IoMT framework.

Following the completion of the approval process, the block will be placed to the queue, at which time it will be assigned an identification in the form of an ID number and a concealed key that cannot be replicated will be generated. After having been sent earlier, the ID and key are then received by the PDA device where they were previously delivered. Using the key, the PDA device encrypts the real medical data and then sends the encoded data, together with the ID and hash of the health data, to the cloud-based database server so that it can be further processed. The ID and hash of the health-data are used in the verification process to ensure that the data are genuine. If someone makes an attempt to corrupt the data stored in a single block, then that attempt will have an impact on the data stored in the blocks that follow it in the chain. After the data has been recognised by using the ID and decoding has been completed by using the key, certain medical institutions may make a request to access medical information that has been compiled and stored in the cloud storage. This request could come after the data has been recognised using the ID. Patients will gain access to the previously encrypted medical information as soon as the decoding procedure is finished. As a result of the fact that a great number of apps for medical care that are powered by deep learning employ this answer as the default, it is essential to be familiar with it. In the realm of healthcare, deep learning systems have a great many different applications that they may do. Several of these applications, such as Medical Assessment Provision, Modified Managements, and Predictive Healthcare, amongst others, have been discussed in the past. The fact that this architecture facilitates the safe transfer of data across a large number of health institutions and that the data may be used in a diverse collection of applications that are pertinent to the field of medical care is the primary advantage that this architecture provides. In our design, the prerequisites for adequate safety have been satisfied. Authenticated users are the only ones who can see and save data, whereas all other users are unable to do so.  Figure 12 shows the IoMT framework.

Techniques using cryptography are helpful in the safeguarding of confidential information. Both the design of the block-working chain and the fact that the access-regulator is centred on a secret key contribute to the fact that the system's integrity is not compromised in any way. Due to the fact that the hidden key can only be created by the nodes of the block chain, information can only be decrypted by those nodes. It is also important to note that basic safety measures contributed to the value of IoMT and cloud-supported medical care structure advantages in order to prevent other calculating difficulties that result in extra resource consumption as a result of the independent execution of encryption algorithms. This is important to note because it is important to note that basic safety measures contributed to the value of IoMT and cloud-supported medical care structure advantages. If the strategy of encrypting information at the source and then decrypting it at the matching destination is employed, it will take a lengthy time, which is unacceptable in applications such as health care. This is because medical information is accessible by a large number of parties.

## 4. Analysis with the Experimental Outcomes

Utilizing several learning modules, the framework is constructed inside of an integrated development environment (IDE) written in *Python*. For the objectives of pretraining and evaluation, both LIDC-IDRI scanned images and PET scanned photos are used in conjunction with one another. The platforms that have been used to verify these models are as follows: “NVIDIA JETSON GPU System-on-Module with 256-core NVIDIA Pascal^TM^ GPU architecture and 256 NVIDIA CUDA cores” and “NVIDIA JETSON GPU System-on-Module with 256-core NVIDIA Pascal^TM^ GPU architecture and 256 NVIDIA CUDA cores.” When compared to other models that are presently being used, the model that has been presented has a lower level of temporal complexity. This can be attributed to the model's optimal speed as well as its efficient design. This is due to the fact that the model that was recommended was created. Applying the standard formula, which is presented for your reading in the following part for your convenience, is what is done to determine the performance parameters.

**Figure 13:**
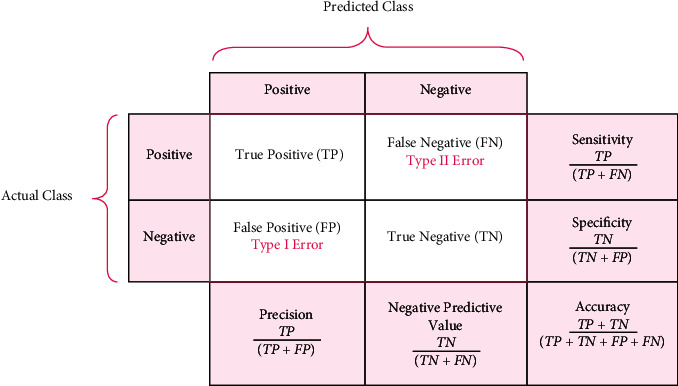
Estimated performance metrics for validating the proposed SLD-SC model.

Figure 13 depicts a comparative comparison of our proposed model with current models, such as the SVM model and the enhanced FCM model, using performance indicators, such as accuracy, sensitivity, and specificity.

**Figure 14:**
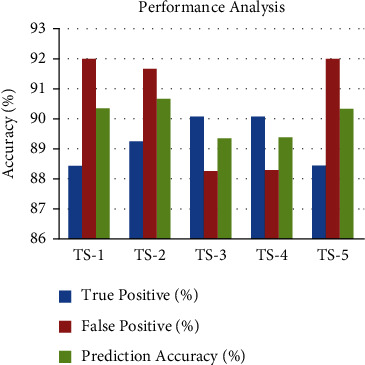
Performance analysis of TP, FP, and accuracy.

In Figure 14, TS-1 to TS-5 describe the types of stages in lung cancer that is predicted correctly and misclassified. It shows that the TS-1 can be found at early stages with 88.56% accuracy rate.

**Figure 15:**
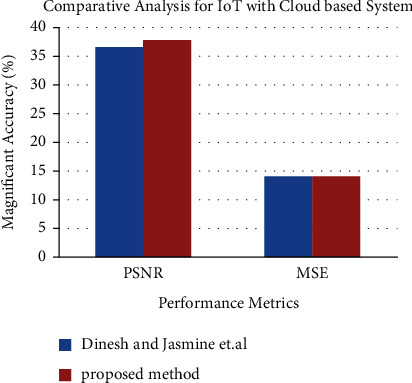
Performance metrics for IoT with Cloud-based system.

Figure 15 shows the results of a comparison between the proposed technique with the performance metrics of existing IoMT techniques are shown in Figure 15. This is as a result of the utilisation of superior lung tumour segmentation techniques for more accurate tumour prediction selection.

## 5. Conclusion

This article focuses on the construction of a lung tumour detector that is based on the Internet of Things in order to decrease the mortality rate that is linked with lung tumours. The suggested SLD-SC module was able to identify the tumour with a higher degree of precision thanks to the use of unsupervised learning neural networks both as a predictor and stage classifier. The LSTM model, which is the most popular Q-learning model, has had its structure updated, and it has been constructed as a lung tumour detector based, among other things, on characteristics retrieved from DICOM and PET scanned pictures. This was accomplished by using the information obtained from those two types of images. On the basis of the segmented photos, which are subsequently fed back into the segmenter, a multilayer CNN model is used to identify and grade each patient's severity level. This information is then sent back into the segmenter. For the segmented images that were put through the evaluation process, an accuracy level of 97 percent in stage categorization was achieved. These deep learning and smart lung tumour models are able to identify tumours at an earlier stage than was previously possible, and this is made possible by the use of superior virtual monitoring and E-diagnosis tools. During the course of our future work, we want to first construct and then analyse the suggested system for huge databases. Following that, we will publish our results about how the system is able to manage vast amounts of hospital data in a secure manner. In addition to that, it is intended that throughout the course of future development, this prototype will be turned into a fully functional Internet of Things hardware. Limitations of the proposed work include only few characteristics that have been retrieved for cancer nodules. No preprocessing like noise reduction and picture smoothing which might possibly help in boosting the detection of nodules correctly has been applied. No categorization as benign or malignant of removed cancer has been conducted [46–49].

## Figures and Tables

**Table 1:** Existing methodology with comparison of the performance metrics.

Paper details	Techniques used in the existing methodology	Datasets available	Accuracy rate of the existing work
Tafadzwa et al. (2021)	Supervised CNN predictor	LUAD	AUC = 71%
Pragya et al. (2021)	SVM, KNN, and CNN	LIDC-IDRI, LUNA 16	Accuracy = 91%
Kalaivani et al. (2021)	Deep CNN model	LIDC-IDRI	Accuracy = 90.85%
Khalifa et al.	BPSO-DT	LUNA	Acc = 88.25%
Abdulgani et al.	TEP classification model	LUAD	Accuracy = 92.65

**Table 2:** Patient ID with stages.

Patient ID	Stage
LUNG1-001	2
LUNG1-002	2
LUNG1-003	2
LUNG1-004	2
LUNG1-005	4
LUNG1-006	3

**Table 3:** LSTM features.

Layer 1	Layer 2	Layer *n*
64	64	64
256	256	256

**Table 4:** Lung tumor stages classified based on its size.

Features	Extracted
Standard deviation	0.12346
Mean	0.24597
Median	0.36798
Entropy	0.46479
Skewness	0.89764

**Table 5:** Estimated classification results for test data.

Images	Classification trained	Classification tested
Img 1	Class 1	Class 1
Img 2	Class 2	Class 2
Img 3	Class 1	Class 1
Img 4	Class 1	Class 1
Img 5	Class 2	Class 2
Img 6	Class 1	Class 1
Img 7	Class 2	Class 1

## Data Availability

The data that support the findings of this study are available at https://www.kaggle.com/datasets/mohamedhanyyy/chest-ctscan-images.
